# Adaptor Molecules Epitranscriptome Reprograms Bacterial Pathogenicity

**DOI:** 10.3390/ijms22168409

**Published:** 2021-08-05

**Authors:** Adamantia Kouvela, Apostolos Zaravinos, Vassiliki Stamatopoulou

**Affiliations:** 1 Department of Biochemistry, School of Medicine, University of Patras, 26504 Patras, Greece; kouvela.a@upatras.gr; 2 Department of Life Sciences, School of Sciences, European University Cyprus, Nicosia 2404, Cyprus; 3 Cancer Genetics, Genomics and Systems Biology Group, Basic and Translational Cancer Research Center (BTCRC), Nicosia 1516, Cyprus

**Keywords:** tRNA, tRNA epitranscriptomics, post-transcriptional modifications, infection, bacterial pathogens

## Abstract

The strong decoration of tRNAs with post-transcriptional modifications provides an unprecedented adaptability of this class of non-coding RNAs leading to the regulation of bacterial growth and pathogenicity. Accumulating data indicate that tRNA post-transcriptional modifications possess a central role in both the formation of bacterial cell wall and the modulation of transcription and translation fidelity, but also in the expression of virulence factors. Evolutionary conserved modifications in tRNA nucleosides ensure the proper folding and stability redounding to a totally functional molecule. However, environmental factors including stress conditions can cause various alterations in tRNA modifications, disturbing the pathogen homeostasis. Post-transcriptional modifications adjacent to the anticodon stem-loop, for instance, have been tightly linked to bacterial infectivity. Currently, advances in high throughput methodologies have facilitated the identification and functional investigation of such tRNA modifications offering a broader pool of putative alternative molecular targets and therapeutic avenues against bacterial infections. Herein, we focus on tRNA epitranscriptome shaping regarding modifications with a key role in bacterial infectivity including opportunistic pathogens of the human microbiome.

## 1. Introduction

The misuse of antibiotics, along with the lack of novel and specific antibacterial agents development, has turned common bacterial infections into major health threats. Bacterial infection results from the invasion in the host and the rapid proliferation and spread of the pathogen [1]. The transition from the bacterial invasion to the host pathogenesis requires the expression of specific virulence factors and the prompt adaptation of the pathogen to multiple external stimuli. Such signals may include the pathogen evasion of the host immune system and antibiotic factors, as well as the encounter of stress environmental conditions that may include elevated temperature or rapid changes in pH [2]. Therefore, the pathogen undergoes a stringent control of its gene expression at multiple levels where coding as well as non-coding RNAs play a pivotal role [3]. RNA-mediated mechanisms with regulatory roles in diverse processes, including chromatin remodeling, ribosomal RNA (rRNA) biogenesis, cell cycle arrest, and even resistance or susceptibility to antibiotics, have been widely characterized in all three domains of life [4,5,6]. In addition to the expression, the post-transcriptional editing of such non-coding RNAs is a key player affecting RNA functionality. This kind of RNA decoration altogether constitutes the epitranscriptome of each RNA species and is largely implicated in the regulation of multiple distinct and interconnected biological processes [7,8,9].

Epitranscriptomics is a new scientific field, which emerged due to the rapid progress of high throughput technologies that gave the ability to acquire and manipulate large datasets [10,11]. Most of the post-transcriptional modifications have a dynamic behavior and affect specific RNA functions, while others are exclusively detected in certain organisms, indicating the existence of a “species-specific code” that had, until recently, been invisible [12,13]. The number of modifications detected in small non-coding RNAs, including microRNAs (miRNAs), small nuclear RNAs (snRNAs) and small nucleolar RNAs (snoRNAs), is still limited, while few modified residues have been characterized on mRNA molecules. The majority of the known modifications have been detected in the rRNAs and transfer RNAs (tRNAs), which correspond to the most intracellularly abundant RNA molecules [12,14].

Although tRNAs were traditionally considered passive amino acid carriers to ribosomes during mRNA decoding, recent evidence has suggested tRNAs as adapting signaling molecules exhibiting non-canonical functions in bacterial growth and pathogenicity [15,16]. We now recognize that not only the composition of the tRNA pool, but also the tRNA epitranscriptome, is essential for an accurate translation and modulation of the gene expression upon specific environmental cues [3,17]. In addition, approximately 25% of each individual tRNA is chemically modified, making tRNA the most highly post-transcriptionally modified molecule in the cell, either eukaryotic or prokaryotic [18]. Such tRNA modifications may include simple methylations, sulfurations (S), or pseudouridylations (Ψ), which represent the most common modified moieties, but also more complex ones, such as queuosines (Q) [19,20]. Although some tRNA modifications are universal in tRNA pools, such as 5-methyluridine at position 54 (m^5^U54) and Ψ55, the modification pattern can vary even between tRNA isoacceptors [21].

The significance of post-transcriptional modifications relies on their involvement to ensure the proper tRNA folding and stability, the tRNA recognition by aminoacyl-tRNA synthetases and modifying enzymes, and to impede frameshifting errors. In addition, tRNA modifications affect the interaction with the elongation factor Tu (Ef-Tu), the ribosomes, and mRNAs, while aberrantly modified tRNAs redound to severe alterations of their functionality [22]. Alteration of tRNA 2′-O-methylation (Nm), for instance, that stabilizes bases stacking and dihydrouridine may disturb the tRNA charging with the cognate amino acid and even the translation fidelity [23,24,25].

The position at which the modification is located in the tRNA tertiary structure indicates its function; however, the precise role of only a few of these has been clarified until now. Post-transcriptional modifications adjacent to the anticodon stem-loop are predominantly associated with the decoding properties of the tRNA, whereas others affect the stability of the tRNA. The most highly and divergent modified sites of a tRNA molecule are the nucleosides at positions 34 and 37, which are required for ample translation and have been strongly related to the bacterial infectivity [17,26,27,28]. Hypomodification of either of these positions leads to a shift in the translational reading frame and results in the expression of aberrant proteins [29]. Furthermore, hypomodification of nucleosides outside the anticodon loop has been correlated with bacterial sensitivity under environmental stress [17,30]. Moreover, the modification status of tRNAs has a great effect on defining the tRNA-derived small RNA (tsRNA) repertoire, which can exist under physiological or stress conditions and become involved in multiple processes, such as translation regulation and even cross-kingdom communication [31,32,33]. Specific bacterial toxins such as colicin E5 and D promote the cleavage of bacterial tRNAs, resulting in the production of tsRNA halves; however, the addition of 2′-O-methylation at the cleavage site prevents tRNAs from being fragmented by ribotoxins [34,35]. The role of tRNA modifications on modulating the tRNA fragmentation process was recently validated by the development of PANDORA-seq (panoramic RNA display by overcoming RNA modification aborted sequencing) methodology, which, by enzymatically removing RNA modifications, detected novel tsRNAs that had previously been unidentified due to their extensive modification state [36].

Overall, existing data indicate that alterations in tRNA post-transcriptional modifications can affect the bacterial homeostasis. Herein, we summarize the tRNA modifications that impact on the bacterial physiology in response to different cellular demands. Furthermore, we focus on tRNA modified moieties that regulate the bacterial pathogenicity and infectivity, and on the current methodologies that have been advanced and combined to facilitate the shaping of the tRNA epitranscriptome. Nowadays, increasingly advanced high-throughput techniques appear as promising tools to identify new and even species-specific tRNA post-transcriptional modifications. Such efforts will provide the knowledge and the perspective to develop new molecular targets to attenuate the bacterial infectivity and novel effective antibiotics or vaccines to combat the global issue of the antibiotic resistance.

## 2. tRNA Epitranscriptome into the Bacterial Infectivity

For many years, it was considered that tRNA modifications were static and the modified tRNA was passively degraded. However, from studies of stress stimuli emerged the existence of specific “writers” and “erasers” that actively add and remove, respectively, specific modifications responding to environmental cues [37]. Environmental stressors can affect the tRNA epitranscriptome either directly and non-specifically [38,39] or by regulating the expression and function of specific tRNA-modifying enzymes [33]. Considering the conditions that a pathogen is experiencing, the essentiality of many tRNA modified nucleotides can vary [40]. Under normal conditions, the expression of proteins with abundant codons and their cognate tRNAs is increased, while, under stress, the expression of factors bearing specific rare codons is preferred [27]. In the same line, the expression of related modifying enzymes follows the differential expression pattern of tRNAs [41,42]. So far, several “writers” have been detected in bacteria. Some of them are involved in the synthesis of a single tRNA modification, while others participate in either the synthesis of modifications at different positions, or require the cooperation of additional protein factors [43]. 

Therefore, in *Escherichia coli*, different members of pseudouridine synthases are implicated to pseudouridylate different positions of a tRNA molecule, such as the tRNA pseudouridine synthases A and B (TruA and TruB) and the dual-specificity RNA pseudouridine synthase RluA, which modify nucleosides at positions 55, 38–40, and 32, respectively [44]. In addition, TrmD is a totally conserved methyltransferase across bacteria that modifies 1-methylguanosine at position 37 (m^1^G37) and is essential for bacterial growth [45], while different methyltransferases deposit methylations elsewhere on the tRNA, including TrmJ (Cm32, Um32) and TrmB (m^7^G46) [46,47]. Moreover, sulfurations occur at positions 8, 32, 34, and 37 of bacterial tRNAs and in position 54 of thermophilic bacteria, which are, respectively, modified by the writers IscS, MnmA, TtcA, ThiI, and TtuA [48]. Dihydrouridines are abundant in bacterial tRNAs and are synthesized by the tRNA-dihydrouridine synthases DusA, DusB, and DusC [49]. In contrast to their eukaryotic counterparts, TsaB and TsaE, which synthesize threonylcarbamoyladenosine (t^6^A) in nucleoside 37 of bacterial tRNAs, are essential for bacterial viability [50]. In addition, tRNA(Ile)-lysidine synthase (TilS) is a bacterial-specific enzyme that catalyzes the modification k^2^C34 of the tRNA^Ile^ [51], and TmcA produces the N4-acetylcytidine (ac^4^C34), which is essential for translation fidelity in *E. coli* [52]. Furthermore, bacterial Q34 is synthesized de novo from a cascade of enzymes, whereas, in eukaryotes, G34 is just replaced with queuine [53]. Recently, it was reported that TadA is responsible to deaminate adenosine 34 to inosine, and the enzymes MiaA, TrmL, and TusA to synthesize the isopentenyl-N6-A37 (i^6^A37) in *E.coli* [54]. Finally, the most recently identified modifying enzyme in *E. coli* is the tRNA aminocarboxypropyltransferase (TapT) and was found to produce the acp^3^U modification in tRNA [55]. 

On the other hand, and despite ALKB, which catalyzes the oxidative demethylation of m^1^A, m^3^C, and m^1^G of both bacterial RNA and DNA, and ms^2^C of specific tRNAs, eraser enzymes that act exclusively on tRNAs have not been identified in bacteria so far [56]. In contrast, the mammalian ALKB homolog ALKBH1 was the first “eraser” identified to specifically demethylate m^1^A58 of human tRNAs under glucose deprivation [57]. Specifically, it was shown that overexpression of ALKBH1 adjusts not only the levels of tRNA^iMet^ impeding translation initiation but also the ribosome’s tRNA usage, resulting in the reduction of the protein synthesis rate.

Altogether, it is clearly indicated the dynamic and regulatory profile of the tRNA editing, which calls for a thorough investigation on the implicated modifying enzymes. The existing knowledge of bacterial “writers” has notably contributed to the association of tRNA modifications with the bacterial pathogenesis, and the advance of hypersensitive technologies able to capture all modifications in each tRNA is expected to elucidate the regulatory role of the tRNA epitranscriptome.

### 2.1. tRNA Modification at the Wobble Position 34

One of the most frequently modified positions on both the bacterial and the eukaryal tRNAs is the nucleotide 34, the so-called “wobble position”. Of note, position 34 can bear diverse post-transcriptional modifications, which each individually alter the tRNA properties in order to ensure an accurate translation. The wobble position of the bacterial tRNA^Gln^, tRNA^Lys^, and tRNA^Glu^ (U34) is usually decorated with a conserved 5-(carboxy)methylaminomethyl-2-thiouridine ((c)mnm^5^s^2^U34). A lack of this modification in *Salmonella enterica*, the pathogen that causes salmonellosis in human, reduces the growth rate at low temperatures (Table 1) [58]. The addition of a 5-carboxymethylaminomethyl (cmnm^5^) group at U34 of the bacterial tRNAs is directed by GidA and MnmE, which act synergistically in *E. coli*, as well as in *S. enterica*. Both enzymes are proposed to influence bacterial physiology and pathogenicity, while they exhibit distinct roles depending on the bacterial species [59]. The expression of virulence factors in *S. enterica* serovar Typhimurium (STM) that lack GidA activity was downregulated reducing the cytotoxicity in host cells and inhibiting the disease onset [60,61]. The immunization of mice with GidA-deficient STM strains protected the mice from subsequent infection by the wild type STM. The same report describes that Salmonella mutant strains lacking GidA or both GidA and MnmE activities exhibited a reduced ability to invade intestinal epithelial cells by 1000-fold, whereas a MnmE-deficient strain was reduced by approximately 100-fold compared to the wild type strain. Interestingly, the invasion ability of the GidA and MnmE mutants was restored after *gidA* and *mnmE* complementation [62].

Cytotoxic necrotizing factor 1 (CNF1) is an A-B bacterial toxin that is considered a virulence factor for meningitis caused by *E. coli* strains, since CNF1 enables *E. coli* K1 strains to invade into the blood–brain barrier endothelial cells. Interestingly, the GidA-modifying enzyme affects CNF1 expression, and knock out of GidA led to a reduction in CNF1 levels [63]. Although the exact mechanism of CNF1 downregulation is not clear yet, it seems that CNF1 translation is mRNA context-dependent. Therefore, the existence of codons in specific CNF1 mRNA regions probably requires GidA-modified tRNAs to be decoded, and GidA deficiency leads to the inhibition of CNF1 translation [63].

In addition, GidA affects the virulence of *Streptococcus mutans*, which naturally colonizes the human oral cavity causing tooth decay. Hence, GidA deletion reduced the bacterial survival under stress conditions, such as acidic pH, the main virulence factor for *S. mutans*, elevated temperature, high osmotic pressure, and the presence of bacitracin [64]. Strikingly, many virulence factors such as SpeB are found downregulated in the absence of GidA in *Streptococcus pyogenes*, the pathogen that is responsible for a variety of human diseases, including pharyngitis, impetigo, and rheumatic heart disease and scarlet fever. Previous studies have proposed that mutants deficient for the pathway of GidA-mediated tRNA modification could be a promising tool to construct a vaccine against *S. pyogenes* [65]. Moreover, GidA deficiency led to reduction in the hemolytic and cytotoxic activity of the enterotoxin Act, a potent virulence factor of *Aeromonas hydrophila* [66]. 

The expression of virulence factors in *Shigella flexneri*, whose colonization results in gastrointestinal disorders and, most commonly, diarrhea in humans, depends on two specific tRNA modifications: first, the Q34 at the wobble position of tRNA^Tyr^, tRNA^His^, tRNA^Asp^, and tRNA^Asn^ that is synthesized by a cascade of enzymes, including the queuine tRNA-ribosyltransferase (Tgt); and second, the 2-methylthio-N6-isopentenyladenosine at position 37 (ms^2^i^6^A37) that is synthesized by the MiaA dimethylallyltransferase and deposited on tRNA species that read codon triplets starting with U, except for tRNA^Ser^. Of note, the absence of either of these modifications downregulated VirF, a transcriptional activator that regulates the expression of many virulence genes, leading to a reduced virulence of the bacterium [67,68].

### 2.2. ROS-Induced tRNA Modifications

Reactive oxygen species (ROS), such as hydrogen peroxide (H_2_O_2_), hydroxyl radical (^•^OH), and superoxide (O_2_^−^), are generated as byproducts of biological procedures, including cellular apoptosis and protein phosphorylation, and their accumulation can lead to oxidative stress. Although the response of eukaryotes to oxidative stress is well-studied at translational level, the underlying mechanisms in bacteria are still obscure. It has been reported that hypoxia in *Mycobacterium bovis* BCG altered 40 modified nucleotides on tRNA molecules, which regulated the expression of many genes. Notably, these changes are so dynamic that they exhibit a differential profile upon different stages of hypoxia, causing codon-biased translation and deregulation of the DosR regulon. Genes encode specific antigens during the latent tuberculosis infection and are pivotal for the bacterium persistence in hypoxia. Specifically, the level of 5-oxyacetyluridine at position 34 (cmo^5^U34) on tRNA^Thr(UGU)^ was increased in early hypoxia over 350%, while the levels of the isoacceptor tRNA^Thr(GGU)^ and the proteins enriched with the cognate codon ACC were decreased (Table 1) [27]. 

Another study proposed that the N6-methyladenosine at A37 of tRNA_1_^Val^ in *E. coli* is produced by the methyltransferase YfiC, and though it is considered a nonessential modification for the bacterial growth under normal conditions, it seems to promote the bacterial survival under osmotic and oxidative conditions [40]. In addition, TrmJ, the enzyme that catalyzes the formation of cytidine(32)/uridine(32)/adenosine(32)-2′-O-methylationwas found to protect *P. aeruginosa*, an opportunistic pathogen associated with pneumonia and sepsis syndromes, against H_2_O_2_-induced oxidative stress. In the same study, catalase, one of the main ROS tolerant enzymes that bacteria use to overcome oxidative stress, was found reduced in a TrmJ mutant, while it was recovered after TrmJ complementation [46]. Besides TrmJ, TrmB, the methyltransferase that catalyzes the m^7^G46 on tRNAs, seems to play a role in bacterial persistence under oxidative stress. In response to H_2_O_2_, m^7^G46 levels, as well as the genes *katA* and *katB* that encode the catalase were increased. In the absence of TrmB, the expression level of *katA* and *katB* genes was reduced, and the bacterial strain was more sensitive in the presence of H_2_O_2_ [47].

Under stress conditions, TtcA, the enzyme that catalyzes the tRNA thiolation (s^2^C32) through an iron-sulfur ([Fe-S]) cluster, was upregulated, whereas a *P. aeruginosa* ttcA-deleted strain was hypersensitive in the presence of H_2_O_2_. The infection of *Drosophila melanogaster* with the *P. aeruginosa* ttcA mutant strain failed, indicating that a TtcA enzyme is also required for an efficient virulence [69]. In addition, mutations in genes that participate in the 2-thiolation of mnm^5^s^2^U in *E. coli* K-12 unsettled the intracellular redox state, leading to deregulation of the respiration activity, the ATP/ADP ratio, the nucleoside triphosphate levels, the DnaA activity, and of other enzymes involved in the cellular respiration status [70].

### 2.3. Membrane-Associated tRNA Modifications

In a series of recent studies, it was revealed that the m^1^G37 modification on tRNAs is required for the expression of many membrane proteins, such as LolB and OmpA, which are necessary for the construction and stability of the outer membrane in Gram-negative bacteria. The m^1^G37-tRNA modification controls the translation of proline codons, specifically CC[C/U] which are essential for the polypeptide junctions in these membrane-associated proteins (Table 1). In addition, it was demonstrated that a lack of TrmD, the writer of the m^1^G37-tRNA modification in *E. coli* and *S. enterica*, led to the disruption of the outer membrane structure and an increase in bacteria sensitivity in antibiotics. In the absence of the modification, stalling and +1 frameshifting occurred, resulting in the premature termination of translation. Given that LolB and OmpA can provide multidrug-resistance, m^1^G37-tRNA modification could be considered as an alternative target to sensitize bacteria against antibiotics [71,72,73,74].

### 2.4. Temperature-Related tRNA Modifications

Bacteria that need to adapt in extreme temperature conditions harbor more tRNA modifications in order to increase the tRNAs melting temperature and preserve their structure stability. For instance, mammalian pathogens have to deal with the elevated temperature of the host during infection. In humans, the average body temperature is 37 °C but it can rise up to 41 °C as a result of the immune response during infection; thus, pathogens have to adapt at these temperatures in order to proliferate [17]. On the other hand, low temperatures decrease enzymatic activity, metabolism rates, and membrane viscosity; thus, bacteria need adaptive mechanisms to survive at such conditions as well. The mutation of *truB* gene, which codes for the writer enzyme of pseudouridine (Ψ), leads to deficient Ψ55 tRNA in *E. coli*, and results in inefficient growth and survival of bacteria at temperatures higher than 37 °C (Table 1) [75]. On the contrary, studies in *Thermus thermophilus* pointed out that a lack of Ψ55 in tRNAs leads to reduced growth acceleration at low temperatures. This is associated with the simultaneous aberrant increase in Gm18, m^5^s^2^U54, and m^1^A58 tRNA modifications [76]. The lack of the TrmI enzyme that modifies m^1^A58 also led to a thermosensitive phenotype [77]. Furthermore, additional modifications are required for cell viability at elevated temperatures. It has been revealed that 7-methylguanosine at position 46 (m^7^G46) of tRNAs is required for the extreme thermophilic *T. thermophilus* viability at high temperatures. A lack of the bacterial tRNA methyltransferase TrmB, which introduces m^7^G46, affects the modification of other nucleotides as well, resulting in the downregulation of protein synthesis and reduced growth rates of the bacterial strain at 80 °C [78]. In the same line, strains lacking the 2-thioribothymidine (s^2^T) at position 54 of tRNAs exhibit a sensitive phenotype at high growth temperatures [79].

## 3. Methodologies for Detection of tRNA Modifications

Despite the fact that the field of the RNA editing has drawn scientific interest, the tRNA epitranscriptome shaping involves many difficulties and remains highly challenging. Although traditional approaches, such as two-dimensional thin-layer chromatography, can identify individual modifications, they are time-consuming and have been replaced from novel high resolution methodologies [80]. The development of specific antibodies against RNA modifications enabled the advance of methodologies that are widely used to map RNA modifications, such as RNA immunoprecipitation (RIP) and cross-linking and immunoprecipitation (CLIP) [81]. Currently, the most widely used approach to analyse the epitranscriptomics profile of tRNAs is based on a combination of tRNAseq followed by a high-resolution liquid chromatography coupled with tandem mass spectrometry (LC-MS/MS) [82].

The development of high-throughput RNA sequencing has provided an alternative approach for the identification of tRNA modifications (tRNAseq). The method’s principle includes reverse transcription (RT) of tRNAs, and different applicable protocols have been optimized to overcome the reverse transcriptase halt that occurs when it approaches to specific modifications causing premature RT stop or nucleotides misincorporation. DM-tRNAseq protocol utilizes a template-switching and thermostable reverse transcriptase (TGIRT) and employs the enzymatic removal of base modifications in order to overcome the bias in tRNA cDNA libraries resulted from reverse transcription step [83]. Hydrolysis-based tRNA sequencing (hydro-tRNAseq) is another tRNA sequencing method, which includes tRNA fragmentation with alkaline hydrolysis before library preparation in order to overcome sequencing hurdles [84]. Modification-induced misincorporation tRNA sequencing (mim-tRNAseq) is a newly described strategy, which provides both increased sensitivity and accuracy using a TGIRT enzyme in order to construct a full length cDNA library, without any prior tRNA treatment, in combination with a customized computation analysis [85]. Alternatively, nanopore MinION has been very recently developed to directly sequence tRNAs without employing the reverse transcription and amplification steps. In contrast to the abovementioned methods, nanopore sequencing permits full length tRNA sequencing in combination with the detection of all tRNA isoacceptors from an RNA mixture sample, which allows the identification of the modified nucleotides [86].

On the other hand, LC–MS/MS is a high-throughput methodology that includes several steps and provides data for the identification of each individual modification regarding the retention time, molecular mass, and fragmentation patterns [87,88]. The advantage of LC-MS/MS is that it can characterize the entire repertoire of modifications on purified tRNA molecules at once. This approach was recently used to study the tRNA epitranscriptome in *Vibrio cholerae*, revealing a new species-specific modification, m^1^A22 in tRNA^Tyr^, a new tRNA modification, acacp^3^U, and the existence of a new RNA editing process, the C-to-Ψ conversion [89]. In an effort to increase the sensitivity of LC-MS/MS, the ultra-performance LC (nanoLC) technique has been developed, which was initially used to map tRNA modifications in *Staphylococcus aureus* [90]. Unlike other established MS-based RNA mapping methods, a novel and NGS-independent methodology, termed mass spectrometric (MS) ladder complementation sequencing (MLC-Seq), has been very recently described, relying on the construction of high quality tRNA ladders and the use of advanced algorithms. Without the requirement of prior NGS data, MLC-seq allows for the direct sequence of full length tRNA molecules and the simultaneous identification of different tRNA species and the contained modifications, overcoming the obstacles incorporated with the cDNA synthesis during the standard NGS-based methodologies [91]. 

Τhe development of increasingly sophisticated methodologies and the combination of state-of-the-art techniques highlights both the challenge and the importance of systematic mapping and quantifying all tRNA modifications. Nonetheless, an epitranscriptome analysis should always be followed by wet lab experimental approaches combined with customized bioinformatic pipelines to validate the acquired datasets.

## 4. Conclusions

**Figure 1:**
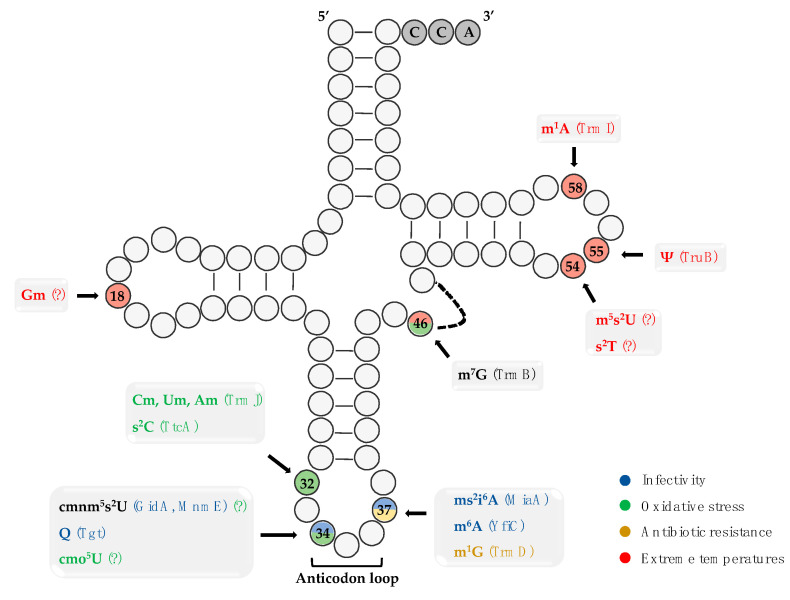
Schematic representation of the tRNA secondary structure and the post-transcriptional modifications that affect the bacterial infectivity and stress adaptability. Blue indicates modifications that affect bacterial virulence, yellow modifications that provide antibiotic resistance, green and red modifications are associated with bacterial adaptation under oxidative stress and extreme temperatures, respectively. Black corresponds to dual-functional modifications. The “writer” enzymes are included in parenthesis.

Post-transcriptional modifications of tRNAs have emerged as pivotal modulators of the gene expression regulation, not only in eukaryotes and yeasts, but also in bacteria. Besides the role of the modified moieties in the maintenance of the tRNA structural stability and the codon usage and translational fidelity, many studies have revealed their contribution to the regulation of bacterial infectivity under stress environmental conditions (Figure 1). 

During infection, pathogenic bacteria have to adapt in external stimuli triggered by the infected host and simultaneously to induce the expression of specific virulence factors. Specific modifications stabilize the structure of the bacterial tRNAs, ensuring the mRNA codons and ribosome recognition, and modulating the tsRNA biogenesis and function. Therefore, the proper modified tRNAs and the alterations occurred upon environmental stressors have been associated with bacterial virulence or resistance. For instance, the main virulence regulator of *S. flexneri*, VirF, is regulated by the presence of Q34 and ms^2^i^6^A37 tRNA modifications [67,68]. In addition, Ψ55 modification enables the survival of bacteria in extreme temperatures [75]. Overall, the implication of tRNA in major bacterial processes, such as protein synthesis and wall and membrane formation, allows specific tRNA modifications to regulate bacterial infectivity either by directly enhancing the translation of virulence factors and virulence-related transcription factors [67,68] or by disrupting the cell wall cohesion and the pathogen survival [71,72,73,74]. However, little is known about the changes in the tRNA modification state and whether this can be the cause of pathogenicity or is simply correlated to the infected state [17]. The surprising fact that the same modification can affect variable factors in different bacteria calls for deeper investigation of the tRNA epitranscriptome in an expanded number of model organisms. Recent technological advances and the wide use of mass spectrometry and sequencing technologies can now contribute towards this approach. Of note, TrmD, a highly conserved and essential methylotransferase in bacteria modifies the m^1^G37 and does not represent any homology with Trm5, the eukaryotic counterpart [92]. Therefore, this specificity has already brought TrmD to the frontline as a potential antibacterial target [93,94]. Furthermore, t^6^A37, another essential modification for many bacteria, such as *E. coli* and *S. aureus*, is not required for other bacterial species or yeasts survival. Moreover, t^6^A37 biosynthetic pathway in bacteria differs from that in eukaryotes and could be also a target for antimicrobial compounds [95]. 

Notably, and apart from the tRNA epitranscriptome direct effect on pathogenic bacteria, it should be further studied in terms of the bacterial microbiome which physiology allows opportunistic pathogens to thrive and infect the host. Moreover, ongoing findings about the role of tRNA modifications and their species-specificity can provide knowledge about novel molecular targets that could be used as tools for antibiotics or vaccines designed against highly resistant pathogens.

## Figures and Tables

**Table 1:** tRNA post-transcriptional modifications with a role in bacterial virulence and adaptability.

Modification	tRNA	Writer	Organism	Function	Reference
**(c)mnm^5^s^2^U34**	tRNA^Gln^/tRNA^Lys^/tRNA^Glu^	GidA/MnmE	*S. enterica*	Regulates virulence factors expression Promotes invasion & salmonellosis onset	[58,59,60,61]
			*E. coli*	Promotes CNF1 expression	[63]
			*S. mutans*	Promotes survival upon acidic pH	[64]
	tRNA^Lys^/tRNA^Leu^/tRNA^Arg^		*S. pyogenes*	Promotes the expression of SpeB, glycohydrolase, streptolysn O, surface M protein, mitogenic factors	[65]
			*A. hydrophila*	Promotes the enterotoxin Act expression Promotes hemolytic and cytotoxic activity	[66]
**cmo^5^U34**	tRNA^Thr(UGU)^	CmoB	*M. bovis BCG*	Regulates DosR regulon	[27]
**Q34**	tRNA^Tyr^/tRNA^His^/tRNA^Asp^/tRNA^Asn^	Tgt	*S. flexneri*	Regulates VirF expression Promotes pathogenicity	[67]
**ms^2^i^6^A37**	tRNA^Axx^	MiaA	*S. flexneri*	Regulates VirF expression Promotes pathogenicity	[67,68]
**m^6^A37**	tRNA_1_^Val^	YfiC	*E. coli*	Promotes survival under osmotic/oxidative stress	[40]
**Cm32** **Um32** **Am32**	tRNA^Met^ tRNA^Trp^ tRNA^Gln^/tRNA^Pro^/tRNA^His^ tRNA^Gln^/tRNA^Pro^	TrmJ	*P. aeruginosa*	Protects against H_2_O_2_-induced oxidative stress	[46]
**m^7^G46**	tRNA^Phe^/tRNA^Asp^	TrmB	*P. aeruginosa*	Promotes bacterial persistence under oxidative stress Upregulates catalase upon H_2_O_2_	[47]
**s*^2^*C32**		TtcA	*P. aeruginosa*	Required for an efficient virulence	[69]
**m^1^G37**	tRNA^Pro^	TrmD	*E. coli*/*S. enterica*	Required for proline codons translation Required for the outer membrane structure Its loss promotes bacteria sensitivity against antibiotics	[70,71,72,73,74]
**Ψ55**	Universal	TruB	*E. coli*	Required for survival over 37 °C	[75]
	Universal		*T. thermophilus*	Regulates Gm18, m^5^s^2^U54 & m^1^A58 tRNA modifications	[76]
**m^1^A58**	tRNA^Asp^	TrmI	*T. thermophilus*	Regulates the bacterial thermosensitivity	[77]
**m^7^G46**	tRNA^Phe^/tRNA^Ile^	TrmB	*T. thermophilus*	Promotes viability at high temperatures Regulates protein synthesis & growth at 80 °C	[78]
**s^2^T54**		TtuA/TtuB	*T. thermophilus*	Promotes viability at high temperatures	[79]

## Abbreviations

VapC	Virulence associated protein C
TrmB	tRNA (guanine-N(7)-)-methyltransferase
TrmD	tRNA (guanine-N(1)-)-methyltransferase
TrmJ	tRNA (cytidine/uridine-2′-O-)-methyltransferase
IscS	iron-sulfur cluster assembly gene
MnmA	tRNA-specific 2-thiouridylase
TtcA	tRNA-cytidine(32) 2-sulfurtransferase
ThiI	tRNA sulfurtransferase
TtuA	RNA-5-methyluridine(54) 2-sulfurtransferase
TsaB, TsaE	tRNA threonylcarbamoyladenosine biosynthesis proteins B and E
TmcA	tRNA(Met) cytidine acetyltransferase
TadA	tRNA-specific adenosine deaminase
MiaA	tRNA dimethylallyltransferase
TrmL	tRNA (cytidine(34)-2′-O)-methyltransferase
TusA	Sulfur transfer protein
ALKB	alkane hydroxylase
ALKBH1	AlkB homolog 1
GidA	glucose-inhibited division protein A
MnmE	tRNA modification GTPase
SpeB	Streptococcus pyrogenic exotoxin B
VirF	virulence regulon transcriptional activator VirF
DosR	dormancy survival regulator
katA, katB	catalase A and B
DnaA	chromosomal replication initiator protein
LolB	lipoprotein localization protein
OmpA	outer membrane protein A
